# Round the Hospitals

**Published:** 1920-05-08

**Authors:** 


### THE MATRONS' AND SISTERS' DEPARTMENT.

### ROUND THE HOSPITALS.

Dr. Addison's great skill in the composition of the first General Nursing Council will be recognised by all who reflect on the variety of interests to be studied apart from actual nursing. The appointments of the Privy Council secured the indispensable legal element in Mr. J. C. Priestley, K.C., who has been chosen as chairman, and that of rural nursing in Lady Hobhouse. The Board of Education supplies a great educational authority in Miss M. J. Tuke, M.A., principal of Bedford College, Regent's Park, London, and provides for the representation of child welfare in the person of the Hon. Mrs. Eustace Hills. The governing bodies of the voluntary hospitals are represented by the Rev. G. B. Cronshaw, chairman and treasurer of the Radcliffe Infirmary, Oxford. The Fever Nurses' Association and fever institutions generally are represented by Dr. Goodall. Public health in the abstract and medical officers of health in particular are represented by Dr. A. Bostock Hill. Asylum interests, together with the Medico-Psychological Association, of which he is President, find their representative in Dr. Bedford Pierce, while Sir T. Jenner Verrall, M.D., who has retired from practice, is the representative of the British Medical Association.

---

At the general meeting of the London Centre of the College of Nursing, held on April 23, a general sense of exhilaration was apparent resulting from the appointment of nine enthusiastic supporters of the College on the first General Nursing Council under the Registration Act. A great advance has been made in the very important matter of providing a residential club in London. A scheme prepared by Miss Pocock was considered and broadly approved. Before any action can be taken, however, it is necessary to consult the various Local Centres of the College, for it is largely for their benefit that the club will exist, and to secure their support their interests must be studied. Moreover, experiments in club management of a very interesting character are being tried in several localities in the kingdom, and the experience thus gained cannot fail to be of value to the London Centre. There is a growing call in London for central commodious premises where members of the College can be received on visiting town, and we believe that the necessary share capital would speedily be subscribed were the undertaking framed on an adequate basis.

---

The Hull Guardians have taken steps to reserve the entire block of the Nurses' Home for the use of the nursing staff. Up till now the rooms of the Resident Medical Officer have been located in this block, an arrangement against which he protested, and moreover, the maternity ward and the labour ward were in the same part of the building, about as unsuitably placed as possible. The medical officer will now be permitted to provide his own living quarters outside the institution, and his salary has been raised in consequence to £700 a year. The nursing staff will be rendered far more comfortable by the additional rooms placed at their disposal, and the wonder is that the change was not made before.

---

It is evident that the case of midwives and maternity nurses will need special regulations under the Eight Hours Bill. It will certainly be a great calamity to the members of this hard-worked section if they are excluded from the protection afforded by the Bill to other women in the practice of their profession. Of course, it is impracticable to limit their attendance on their patients to a rigid eight hours. But it should be provided that they obtain their forfeited rest afterwards, and regulations to ensure that this is done could surely be made. The supposed necessity for passing without pause from one case to another, often in a condition of nervous fatigue under prolonged shortage of sleep, must give way to more humane conditions. If it is bad for nurses it is doubly bad for mothers, who need, before all else, the calm and cheerful presence of a nurse in the pink of condition, alert and untired.

---

The Nurses' Co-operation had a record year in 1919. With the staff made up to strength, comprising 442 medical and surgical and 36 mental nurses, the gross earnings were £61,059, of which the sum of £57,349 was paid to nurses. The balance of income paid all the working expenses of the Co-operation, including those of the Nurses' Home, which were higher than usual this year owing not only to the increased cost of provisions, wages, &c., but also to extensive repairs and renewals which had been unavoidably deferred during the war years. Never have the nurses earned so much as during this last year, and never have the finances and management been in so thoroughly satisfactory a state. The Nurses' Co-operation, mother of many another at home and abroad, has, in truth, fully justified all the hopes with which it was founded nearly thirty years ago. It mourns Sir Henry Burdett, one of those who helped to found it, with sincere and in many cases personal grief, for many of the nurses were known to him, and his interest in their welfare was shown in many practical ways. They could always be sure of his friendly support when difficulties arose, and he followed their rising fortunes with increasing satisfaction.

---

Health Week is in full progress as we go to press, and in many pulpits and most schools people young and old are being warned that it is the business of every citizen to keep himself out of the C 3 category. The days have long gone by when neglect of health was considered manly, but people still lack the instinct for self-preservation which acts as an inward monitor and scourges the victim

**ROUND THE HOSPITALS—(*continued*).**

of self-indulgence. It would be interesting were doctors plainly to indicate what particular forms of disease necessitating operation can be traced to over-eating, for instance. Another subtle form of self-indulgence, failure to take sufficient outdoor exercise, has a good many complaints, some of them mortal, to its count. The truth is that rich, comfortably housed, leisured folk are a prey to preventable ills fully as much as the ill-housed, the over-worked, and the underfed. The old cynical motto "Take care of No. 1" has a new significance in these days, and should be the motto for Health Week.

Dr. McWhir, medical officer of health for the Berwick Rural District, has been speaking some plain home truths about the locality he serves. There is an appalling disregard for the decencies of life in the overcrowded dwellings of the border country, where young men and women are herded together in one sleeping compartment. He declares that "a cynic who visited our insanitary dwellings might be pardoned for fancying that there is a gigantic conspiracy to supply a constant stream of patients to the costly institutions where consumptives undergo special treatment."

At the thirty-second annual meeting of the Invalid Children's Aid Association, held on April 27, at Londonderry House, Sir William Bennett was in the chair, and a notable gathering assembled. Speeches of a highly interesting character were made by the chairman and by Miss Irene Vanbrugh, the Lord Bishop of Stepney, Major Souttar, and Major Beith (Ian Hay). The work done by the Society is both valuable and extensive, and it covers ground touched by no other organisation. It undertakes the supervision and care of crippled and invalid children after they pass out of hospitals and Poor-Law infirmaries, and its work for the tubercular patients of the L.C.C. is priceless. The number of children dealt with last year totalled 10,247. The Association has now seven homes where open-air treatment is provided and where the children enjoy special educational advantages. They are hoping to open another shortly, should funds be forthcoming.

We are requested to announce that on the Centenary of the birth of Florence Nightingale, Wednesday, May 12, the Dean of Westminster will give an Address at Evensong in the Abbey at 3 p.m. Seats will be reserved for members of the nursing profession. Tickets for these seats may be obtained on application to the Matron, St. Thomas's Hospital, S.E. 1, before May 10, enclosing a stamped addressed envelope.

The foundation-stone of the new Nurses' Home at the Boscombe Branch of the Royal Victoria and West Hants Hospital was laid by the Countess of Malmesbury, C.B.E., on April 22. The funds for the building, which is estimated to cost £14,000, have been provided by Mr. Walter Child Clark. The Home is being built by Messrs. McWilliam and Son, of Bournemouth, to the plans of Mr. H. E. Hawker, F.S.I., M.S.A. Accommodation is provided for thirty nurses, and the building is so constructed as to allow of wings being added at some future date to provide accommodation for a further sixty nurses if necessary.

We note with pleasure the renewed activity taking place in Red Cross divisions in different parts of the country. There is plenty of enthusiasm to build on in furtherance of schemes for raising the condition of the people and both those who are employed during a great part of the day and those who are comparatively idle are to be found among the Red Cross members eager to be of service. At Norwich an important step has been taken in securing at a nominal rent good headquarters for the division where a Red Cross club has been started, membership being confined to members of a V.A.D section. There will be a room available for the important work of training recruits and facilities for study and recreation at a low subscription. At the quarterly meeting of the Herefordshire branch it was reported that a recent call from the dentist headquarters for the enrolment of volunteers had resulted in twenty applications being received. The meeting assented to the proposed amalgamation of the Societies under which members are working, and in future this branch will be styled "The Order of St. John and British Red Cross Society—Herefordshire Branch."

The appeal which is to be launched this week at a meeting held in the hall of St. Bartholomew's Hospital, with Princess Christian in the chair, for a million shillings on behalf of the British Hospital for Mothers and Babies, is worthy of all support. The choice of the Woolwich site for the new institution is a very happy one, and every endeavour will be made, we understand, to raise the standard of midwifery instruction in the training-school attached. It is proposed to have forty-two beds and forty nurses and pupils. The course for trained nurses will last a year, and an extra year's course will be offered to women taking up the career of midwifery teacher. Thus the ideals of Florence Nightingale are likely within a short while to see realisation. She forecasted, it may be recalled, a system under which women trained for two years in midwifery would rank as a special class of midwives or midwifery doctors. The present course is undoubtedly too short, and the intensive system of training it entails makes demands on the endurance of the pupils greatly to be deprecated in the interests of all concerned. The midwifery instructor ought to be, and sometimes is, a woman of education and refinement, but at the present time the inducements offered to such women to take up this branch of work are slight. All who desire to help in inaugurating a better system should communicate with Miss Alice Gregory, Secretary of the movement, British Hospital, Wood Street, Woolwich, S.E. 18.

